# Efficacy of botulinum toxin type A 100 units versus 200 units for treatment of refractory idiopathic overactive bladder

**DOI:** 10.1590/S1677-5538.IBJU.2014.0221

**Published:** 2015

**Authors:** Osama Abdelwahab, Hammouda Sherif, Tark Soliman, Ihab Elbarky, Aly Eshazly

**Affiliations:** 1 Urology department, Faculty of Medicine, Benha University, Egypt

**Keywords:** Botulinum Toxins, Therapeutics, Urinary Bladder, Overactive

## Abstract

**Objective:**

To evaluate the efficacy and safety of a single intra detrusor injection of BoNTA comparing two different doses (100 U or 200 U) in patients with idiopathic overactive bladder.

**Materials and Methods:**

A randomized prospective study evaluated the efficacy of BoNTA in management of refractory idiopathic overactive bladder and included 80 patients. All patients were assessed initially by taking a history, a physical examination, overactive bladder symptom score, urine analysis, routine laboratory investigations, KUB and pelviabdominal. OABSS was adjusted on all patients postoperative at 1,3,6,9 months also Urodynamic was done for all patients preoperative and postoperative at 3, 6, 9 months.

**Results:**

The mean age was 30.22±8.37 and 31.35±7.61 in group I and II respectively. There was no statistically difference between both groups in all parameters all over the study except at 9 months after treatment. Hematuria was observed 6 and 9 patients in group I and II respectively. Dysuria was observed in 6 and 15 patients in group I and II respectively. UTI was detected in 3 and 7 patients in group I and II respectively.

**Conclusion:**

A single-injection procedure of 100 U or 200 U BoNTA is an effective and safe treatment for patients with IOAB who failed anticholinergic regimens. OABSS and QoL were improved for 6 months; 100 U injections seemed to have comparable results with 200 U. There was a significant difference at month 9 towards 200 U with more incidences of adverse events.

## INTRODUCTION

Detrusor overactivity is defined by the presence of lower urinary tract symptoms of urgency with or without urge urinary incontinence (UUI), usually with frequency and nocturia (1). In the majority of affected patients, the cause of the detrusor overactivity is idiopathic while neurogenic detrusor overactivity occurs mainly in patients with spinal cord diseases (2). Conservative treatments (lifestyle modifications, pelvic floor exercises, bladder training, and anticholinergic regimens) may result in insufficient improvements and in low compliance because of bothersome adverse events (3). Intradetrusor injection of botulinum neurotoxin type A (BoNTA) is emerging as the second-line treatment for refractory OAB symptoms (4).

Botulinum toxin is a purified neurotoxin derived from clostridium botulinum and its main effect is to inhibit signal transmission at the neuromuscular junction by inhibiting the release of acetylcholine. In addition, botulinum toxin is now thought to have effects on the release of other sensory neurotransmitters such as substance P and ATP, as well as reducing the axonal expression of capsaicin and purinergic receptors (5). Many studies demonstrated significant improvements in OAB symptoms and QoL with BoNTA treatment but they also showed increased post void residual urine, acute urinary retention and urinary tract infections (6). There is no consensus on the dose of BoNTA or BoNTB, injection sites, and the duration between repeat injections (7). This current study aimed to evaluate the efficacy and safety of a single intra detrusor injection of BoNTA alone comparing two different doses (100 U or 200 U) in patients with IOAB.

## PATIENTS AND METHODS

This study was a randomized prospective one evaluating the efficacy of BoNTA in management of refractory idiopathic overactive bladder (IOAB) and included 80 patients who presented to the Urology Department of Benha University Hospital from May 2011 to February 2014. An informed written consent was obtained from all patients after the study protocol was approved by the Research Ethics Committee, Faculty of Medicine, Benha University. The inclusion criteria were IOAB refractory to previous anticholinergics with different types of anticholinergic agents, either as a single drug or a combination for >3 months. Exclusion criteria were pregnant women, uncorrectable coagulopathies, active UTI, bladder outlet obstruction, neurogenic bladder, or having a PVR>150 mL at the time of enrollment, and previous radiotherapy or antineoplastic treatment. Additional use of anticholinergics was not allowed during the study period. Patients were randomly classified into two groups I and II. They underwent intradetrusor injection of BoNTA 100 and 200 Unit respectively. All patients were assessed initially by taking a history, a physical examination, overactive bladder symptom score (OABSS) (8) (Table 1), EuroQoL (EQ-5D) visual analogue scale (VAS) (9), measuring the patient's current health-related QoL state; both scales range from 0 to 100 (worst to best), urine analysis, routine laboratory investigations, KUB and pelviabdominal spiral CT and IVP if indicated. Urodynamic evaluation was done in the form of flowmetry and cystometry.

**Table 1:** Overactive bladder symptom score.

Question	Frequency	Score
How many times do you typically urinate from waking in the morning until sleeping at night?	≤ 7	0
	8-14	1
	≥ 5	2
How many times do you typically wake up to urinate from sleeping at night until waking in the morning?	0	0
	1	1
	2	2
	3	3
How often do you have a sudden desire to urinate, which is difficult to defer?	Not at all	0
	Less than once a week	1
	Once a week or more	2
	About once a day	3
	2-4 times a day	4
	5 times a day or more	5
How often do you leak urine because you cannot defer the sudden desire to urinate?	Not at all	0
	Less than once a week	1
	Once a week or more	2
	About once a day	3
	2-4 times a day	4
	5 times a day or more	5

Patients were instructed to circle the score that best applied to their urinary condition during the past week; the overall score was the sum of the four scores.

OABSS was developed by Homma et al. (8) which is a single symptom score that employs a self-report questionnaire. There were 4-symptoms evaluated: daytime frequency, nighttime frequency, urgency and urge incontinence for the questionnaire.

The score is the simple sum of the 4-symptom scores.

### Injection technique

After dilution with 10cc saline, either 100 or 200 Units BoNTA (Allergan®, Irvine, CA, USA) were used for cystoscopic intradetrusor injection under spinal anesthesia. The injection was performed in 20 sites, using 30-degree lens and a rigid scope with a 6 Fr. injection needle without side holes (Amecath Company®, Egypt).

The injection sites were determined after mapping of the bladder at the anterior, left lateral, right lateral, posterior walls and the trigone (0.5cc at each site). The injection was followed by insertion of a 16 Fr. Foley's catheter, to be removed the next morning after surgery. All patients received peri-operative I.V. antibiotics. For postoperative follow-up, all patients were assessed at 1, 3, 6, 9 months using OABSS, HRQoL as well as urodynamic study at 3, 6, 9 months.

## Statistical analysis

The collected data were tabulated and analyzed using SPSS version 16 software (Spss Inc.®, Chicago, ILL Company). Categorical data were presented as number and percentages while quantitative data were expressed as mean and standard deviation. Chi square test (X2) and Student “t” test were used as tests of significance.

The accepted level of significance in this work was stated at 0.05 (P<0.05 was considered significant).

## RESULTS

### Demographic baseline values

Eighty patients (63 women and 17 men) were enrolled in the study. The participants were randomly assigned to one of the two treatment groups, receiving a BoNTA dose of either 100 U (n=40) or 200 U (n=40). The mean (standard deviation) ages were 30.22 (8.37) years for group I and 31.35 (7.61) years for group II. One patient who received 100 U of BoNTA dropped out of the study at month 6 evaluation and another one at month 9. Two patients who received 200 U of BoNTA dropped out of the study at month 9 evaluation. There were no statistically significant differences in baseline characteristics between two groups. There was no statistically difference between both groups in all parameters all over the study except at 9 months after treatment.

## EFFICACY (Table 2)

**Table 2:** Clinical symptoms and PVR urine Changes.

Variables		BoNTA 100		BoNTA 200
	N	Mean(SD)	N	Mean(SD)
**Frequency**				
Baseline	40	1.6 (0.496)	40	1.67 (0.525)
At 1m	40	0.45 (0.503)*	40	0.42 (0.5)*
At 3m	40	0.42 (0.5)*	40	0.33 (0.474)*
At 6m	39	0.51 (0.506)*	40	0.3 (0.464)*
At 9m	38	1.1 (0.508)* † ‡ Δ	38	0.32 (0.471)* #
**Nocturia**				
Baseline	40	0.87 (0.965)	40	1.2 (1.202)
At 1m	40	o.23 (0.422)*	40	0.15 (0.361)*
At 3m	40	0.13 (0.334)*	40	0.13 (0.334)*
At 6m	39	0.13 (0.338)*	40	0.12 (0.334)*
At 9m	38	0.36 (0.488)*	38	0.13 (0.342)* #
**Urgency**				
Baseline	40	4.7 (0.464)	40	4.67 (0.474)
At 1m	40	1.4 (1.37)*	40	1.9 (1.12)*
At 3m	40	1.07 (1.163)*	40	1.45 (1.131)*
At 6m	39	0.97 (1.135)* †	40	1.25 (1.031)* †
At 9m	38	2.57 (0.948)* † ‡ Δ	38	1.47 (1.202)* #
**UUI**				
Baseline	40	1.67 (1.899)	40	1.8 (2.002)
At 1m	40	0.77 (1.073)*	40	0.85 (1.098)*
At 3m	40	0.65 (0.975)*	40	0.65 (0.948)*
At 6m	39	0.67 (0.982)*	40	0.72 (1.085)*
At 9m	38	1.26 (1.171)* † ‡ Δ	38	0.68 (0.162)* #
**PVR**				
Baseline	40	25.75 (12.83)	40	27.4 (15.05)
At 1m	40	40.0 (21.42)*	40	47.37 (11.87)*
At 3m	40	39.23 (12.48)*	40	42.00 (10.05)*
At 6m	39	38.88 (12.22)*	40	41.79 (10.77)*
At 9m	38	24.21 (8.58)‡ Δ	38	29.21 (11.30)‡ Δ #

* significant in intragrou *significant in intragroup comparison to “before intervention”

† significant in comparison to “1 month later

‡ significant in intragroup comparison to “3 months later”

Δ significant in intragroup comparison to “6 months later”

# significant in intergroup comparison.

(Paired “t” test was the test of significance)

(N=Number of patients, UUI=Urge Urinary Incontinence, SD=Standard Deviation, PVR=Post Void Residual, M=Month)

### Clinical symptoms

When comparing the mean of OABSS and HRQOL data obtained at months 1, 3, 6 and 9 after treatment to baseline data it was observed significant improvement (p<0.001) in both groups. The mean values of OABSS and HRQOL data at months 3, 6 and 9 were significantly ameliorated (p <0.001) compared to data at month 1 in both groups. Within-group I analyses at month 9 demonstrated a statistically significant amelioration (p<0.001) compared to data at months 3 and 6 (Table 3).

**Table 3:** Urodynamic Changes.

Variables		BoNTA 100		BoNTA 200
	N	Mean(SD)	N	Mean(SD)
**volume at first desire (mL)**				
Baseline	40	200 (35.73)	40	199 (35.71)
At 3m	40	318 (59.62)*	40	300.2 (44.28)*
At 6m	39	309.2 (58.14)*	40	295.5 (40.86)*
At 9m	38	246.8 (53.78)* ‡ Δ	38	291.8 (42.82)* ‡ #
**volume at strong desire (mL)**				
Baseline	40	259 (64.40)	40	260.5 (63.08)
At 3m	40	427.5 (58.78)*	40	407.2 (41.44)*
At 6m	39	417.9 (51.2)*	40	401.2 (38.35)*
At 9m	38	313.1 (67.38)* ‡ Δ	38	392.1 (37.28)* ‡ #
**Detrusor pressure (cm H2O)**				
Baseline	40	27.8 (10.12)	40	30.6 (11.09)
At 3m	40	11.1 (6.317)*	40	9.25 (3.01)*
At 6m	39	10.6 (5.36)*	40	9.07 (3.22)*
At 9m	38	19.2 (7.78)* ‡ Δ	38	10.42 (3.97)* #
**MCC(mL)**				
Baseline	40	277.7 (75.29)	40	289.2 (70.83)
At 3m	40	439 (55.22)*	40	439(41.24)*
At 6m	39	437.4 (55.36)*	40	438.2 (40.99)*
At 9m	38	350 (69.08)* ‡ Δ	38	430.5 (34.24)* #

* significant in intragrou

p comparison to “before intervention”

‡ significant in intragroup comparison to “3 months later”

Δ significant in intragroup comparison to “6 months later”

# significant in intergroup comparison.

(Paired “t” test was the test of significance)

(MCC= Maximum Cystometric Capacity, N=Number of patients, SD=Standard Deviation, M=Month)

### Urodynamic values

Comparison of urodynamic data obtained at months 3, 6 and 9 after treatment to baseline data revealed that the mean of volume at first desire, volume at strong desire, maximal cystometric capacity and detrusor pressure improved significantly (p<0.001) in both groups. The mean values of urodynamic data at month 9 were significantly ameliorated (p<0.001) compared to data at months 3 and 6 in group I.

However, in group II, the mean volume at first desire and at strong desire were significantly ameliorated (p<0.001) compared to data at month 3 only (Table 4).

**Table 4:** OABSS and QOL Changes.

Variables		BoNTA 100		BoNTA 200
	N	Mean(SD)	N	Mean(SD)
**OABSS**				
Baseline	40	8.85 (2.166)	40	9.35 (1.994)
At 1m	40	2.85 (2.537)*	40	3.32 (2.092)*
At 3m	40	2.27 (2.391)* †	40	2.55 (2.417)* †
At 6m	39	2.28 (2.361)* †	40	2.37 (2.518)* †
At 9m	38	5.3 (2.11)* † ‡Δ	38	2.6 (2.307)* † #
**QoL**				
Baseline	40	42.7 (8.58)	40	40.8 (6.82)
At 1m	40	83.6 (7.54)*	40	82.8 (7.60)*
At 3m	40	72.4 (16.45)* †	40	77.3 (11.67)* †
At 6m	39	73.4 (12.21)* †	40	77.3 (10.12)* †
At 9m	38	68.5 (7.57)* †Δ	38	77.1 (10.00)* † #

* significant in intragroup comparison to “before intervention”

† significant in comparison to “1 month later

‡ significant in intragroup comparison to “3 months later”

Äsignificant in intragroup comparison to “6 months later”

# significant in intergroup comparison.

(Paired “t” test was the test of significance)

(N=Number of patients, SD=Standard Deviation, M=Month, OABSS=Over Active Bladder Symptom Score, QoL=Quality of Life)

### Side effects

Early postoperative hematuria was observed in 6 (4 women and 2 men) patients in group I and 9 (6 women and 3 men) patients in group II. During follow-up dysuria was observed in 6 (5 women and 1 male) and 15 (12 women and 3 men) patients in group I and II respectively. UTI was detected in 3 (2 women and 1 male) and 7 (5 women and 2 men) patients in group I and II respectively.

## DISCUSSION

Sacral neuromodulation or surgical bladder augmentation were the available options for treatment of IOAB, however they are highly invasive and have long term complications (10). A European consensus group gave a grade A recommendation for BoNTA use in IDO (4) and a recent systematic review suggested that its use for refractory OAB is well justified (11).

In relation to the aforementioned results, the current study showed significant improvement in all clinical symptoms (frequency, nocturia, urgency and UUI) after BoNTA treatment. We observed no significant difference between 100 U and 200 U at post injection at months 1, 3 and 6. The 200 U BoNTA dosage demonstrated consistent improvements till the end of the study and the significant difference between the study groups was observed at month 9 after injection. However, there was a significant amelioration at month 9 when compared to those at months 1, 3 and 6 in patients who received 100 U except for nocturia. The response rate and the incidence of side effects of BoNTA on IOAB are closely related to the dose (12).

Many investigators reduced the dose of BoNTA to 100 U and a satisfactory outcome can still be achieved for IDO due to the high incidence of side effects after BoNTA injections (7, 11, 13–15). The continence and the cure rate were respectively 22.9% and 15% by Nitti et al. (16), in the series by Visco et al. (17) the cure rate was 27%. In other studies detrusor injections of 200 U yielded long response duration of 12-15 months (12). Brubaker et al. (18) reported a mean duration of efficacy of 370 days of BoNTA 200 U.

The efficacy of a single injection of BoNTA toxin over 6 months and the literature supported 200 U as the dose most likely to provide this durability (19–22). Regarding the changes in the urodynamic parameters after BoNTA injection, we found that the bladder capacity returned gradually, however the maximum effect on significant increase in cystometric capacity was observed at month 3 after treatment in both groups. Although BoNTA remained therapeutically effective up to 6 months, the effect reduced significantly in group I with time till the end of the study.

Doses ranging from 50 U to 300 U showed significantly greater improvement in symptoms of frequency, urgency, and UI as well as in urodynamic measures in the active-treatment arms with BoNTA doses of at least 100 U (18, 19, 23). Regarding the optimal BoNTA dose, one study suggested minimal added benefit above 150 U, (19) and a Class III study comparing 100 U vs. 150 U failed to demonstrate any differences between the two doses (14).

The number of adverse events was lower than the one observed by others. In this study, none of the patients developed urinary retention or significant elevation of post-void residual urine (PVR>100 mL) following injection. Many studies of BoNTA for IDO reported a 24-43% incidence of transient urinary retention requiring CIC and also reported a 32-72% incidence of a large PVR and difficulty in urination (24–28). Although a large PVR and chronic urinary retention remain obstacles for the wide use of BoNTA in treatment of refractory DO, no factors predicting these adverse effects have been found (7). Bauer et al. (29) have specifically looked at adverse events after injection of 100 U, 200 U and 500 U of BoNTA by using a patient questionnaire, but he concluded the higher doses of the toxin led to higher rates of adverse events.

We observed mild hematuria which was procedural related and resolved conservatively. In the series by Kuo et al. (22) the hematuria was up to 7.8% and 3.6% in the series by Chapple et al. (15). In the current study, dysuria was increased in group II. Previous studies also reported dysuria in 5.8% (15), 4% (30) of patients that received 100 U whereas; it was 33% for 200 U (31). On the other hand, many series did not notice this adverse effect (12, 14, 18, 20, 26, 27).

This study revealed more cases of UTI in group II; all cases received proper antibiotics and analgesics, which was comparable with others in which the rate of UTI ranged from 13% to 44% (14, 15, 18, 22, 23, 32) and this was related to clean intermittent self catheterization (CISC).

Our study is not without weaknesses; firstly, this study was done without a control arm. Secondly, it still represents a small number of patients. Finally, from the previous data we claim that further studies will be needed to confirm the effectiveness of 100 U and 200 U doses. They might also consider evaluating the efficacy and tolerability of repeated injections of BoNTA to optimize the risk–benefit ratio. This would help to introduce BoNTA as a treatment of choice in patients with refractory IOAB.

## CONCLUSIONS

A single-injection procedure of 100 U or 200 U BoNTA is an effective and safe treatment for patients with IOAB who failed anticholinergic regimens. Following the procedure, OABSS and QoL were improved for 6 months; 100 U injections seemed to have comparable results with 200 U. At month 9 there was a significant difference towards 200 U with more incidence of adverse events.
